# Supplementary figures and images for: Climate change belief systems across political groups in the United States
Source: PLoS One. 2024 Mar 20;19(3):e0300048. doi: 10.1371/journal.pone.0300048 (PMC10954181; doi:10.1371/journal.pone.0300048)

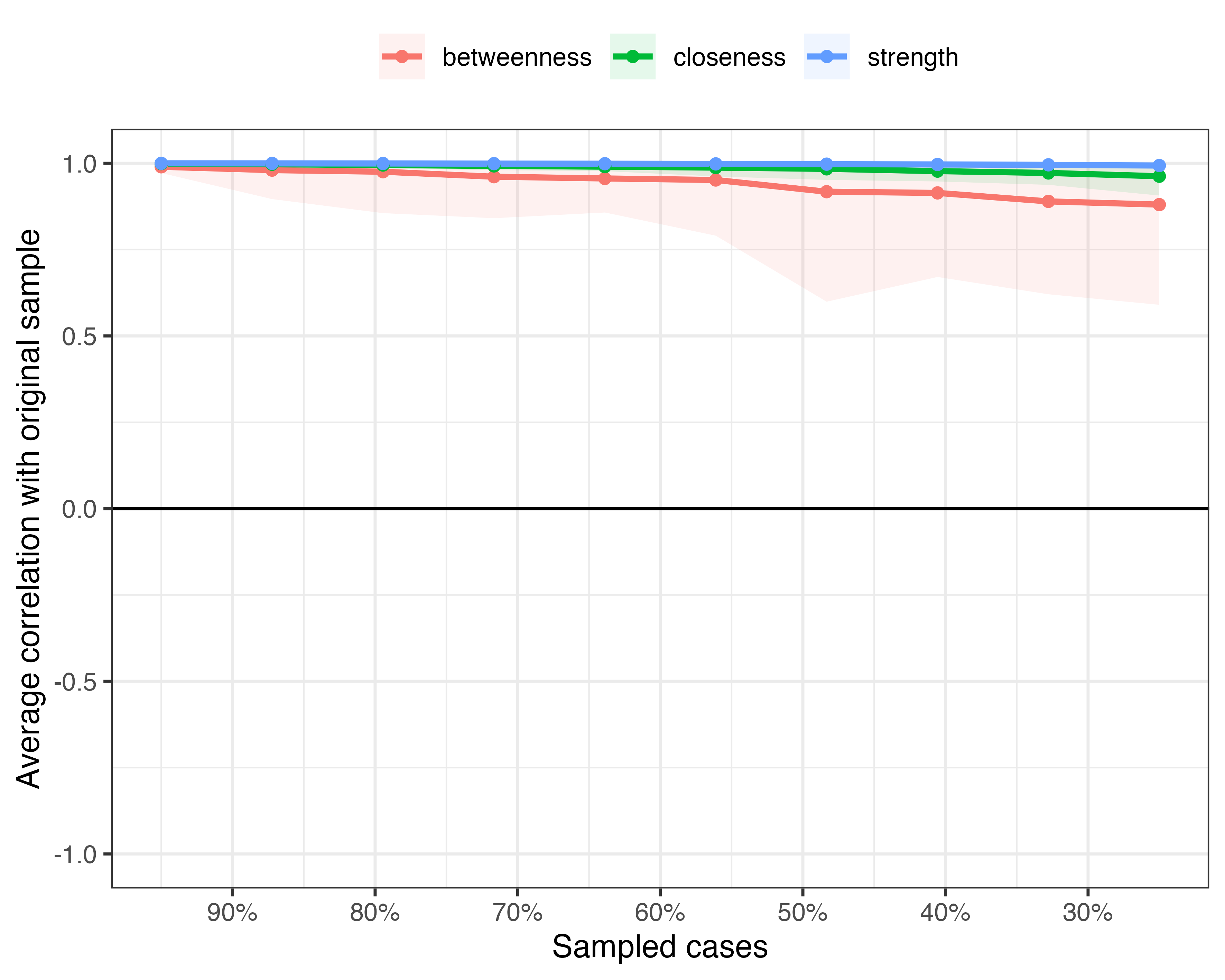

Supplement: S1 Fig — (TIF) [file pone.0300048.s001.tif]

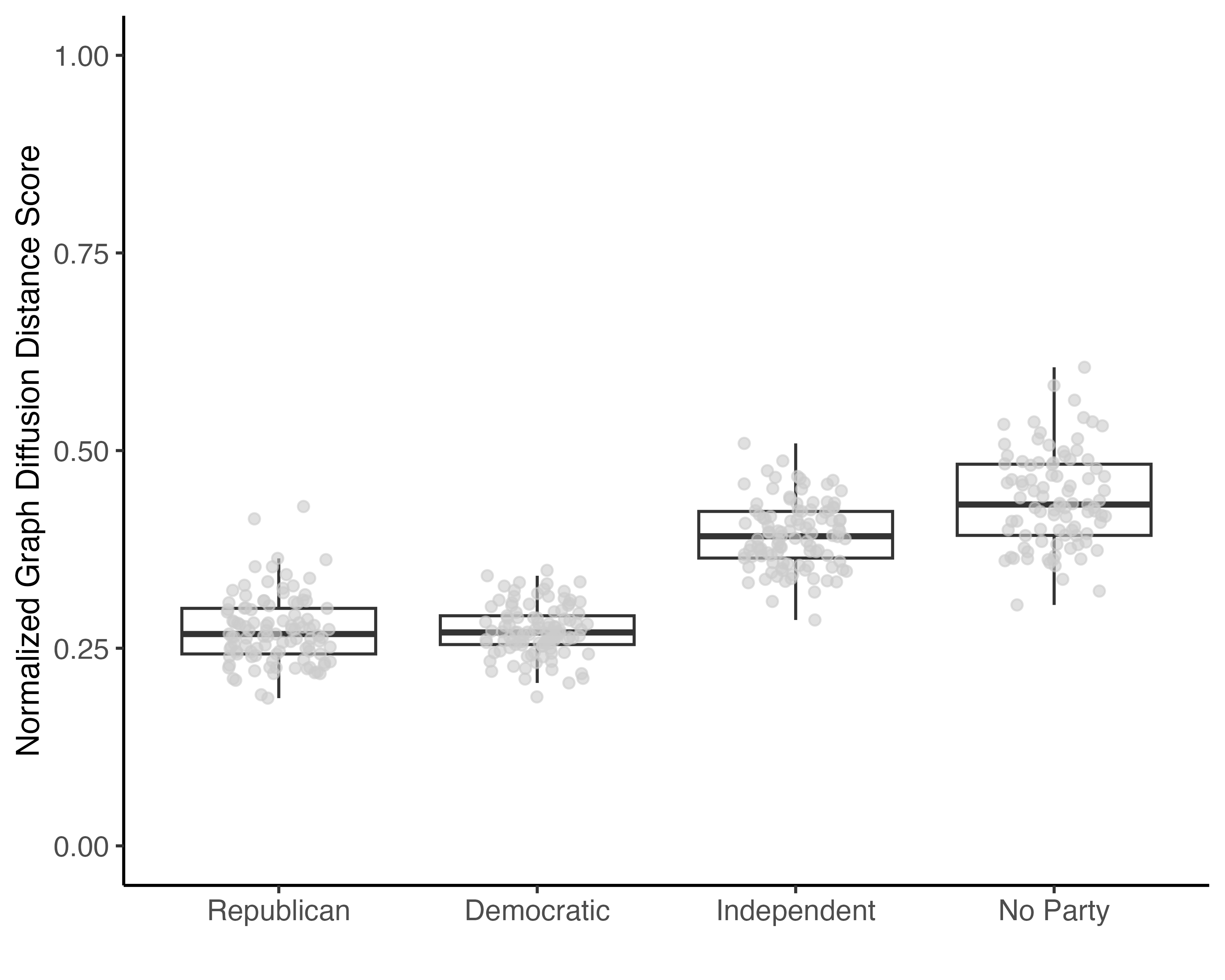

Supplement: S2 Fig — (TIF) [file pone.0300048.s002.tif]

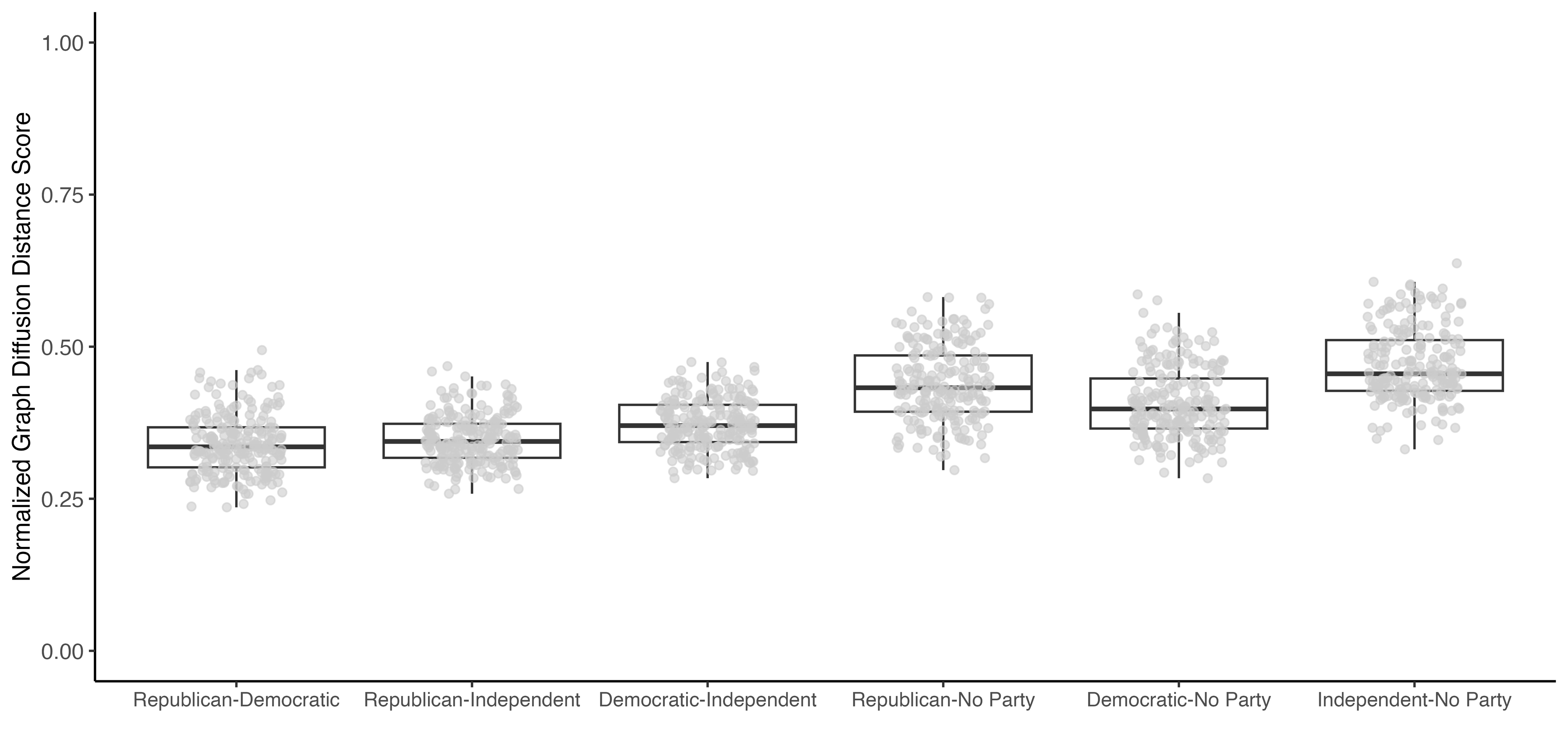

Supplement: S3 Fig — (TIF) [file pone.0300048.s003.tif]
