# Supplementary material for: Climate change belief systems across political groups in the United States
Source: PLoS One. 2024 Mar 20;19(3):e0300048. doi: 10.1371/journal.pone.0300048 (PMC10954181; doi:10.1371/journal.pone.0300048)
Supplement: S1 Table — (DOCX) [file pone.0300048.s004.docx]

**S1 Table. Betweenness centrality difference between worry and other elements**

| Variables | Types | Lower | Upper | Significantly Higher? |
| --- | --- | --- | --- | --- |
| GW happening | Betweenness | 22 | 62 | Yes |
| GW human cause | Betweenness | 28 | 70 | Yes |
| GW consensus | Betweenness | 38 | 76 | Yes |
| Collective efficacy | Betweenness | 40 | 76 | Yes |
| Community risk | Betweenness | 28 | 64 | Yes |
| US risk | Betweenness | 24 | 72 | Yes |
| Risk time | Betweenness | 30.05 | 68 | Yes |
| General attitude | Betweenness | 12 | 72 | Yes |
| Policy support CO_2_ | Betweenness | 8 | 46 | Yes |
| Policy support fund | Betweenness | 26 | 66 | Yes |
| Policy support rebate | Betweenness | 40.05 | 78 | Yes |
| Political behavior | Betweenness | 32.05 | 78 | Yes |
| Consumer behavior reward | Betweenness | 10.05 | 56 | Yes |
| Consumer behavior punish | Betweenness | 22 | 58 | Yes |

*Note*. Betweenness centrality of worry was compared with that of other variables. Lower and Upper indicate 95% confidence interval of the difference. Significance was tested by comparing the difference score with 0 at the alpha level 0.05.
