# Supplementary material for: Climate change belief systems across political groups in the United States
Source: PLoS One. 2024 Mar 20;19(3):e0300048. doi: 10.1371/journal.pone.0300048 (PMC10954181; doi:10.1371/journal.pone.0300048)
Supplement: S2 Table — (DOCX) [file pone.0300048.s005.docx]

**S2 Table. Closeness centrality difference between worry and other elements**

| Variables | Types | Lower | Upper | Significantly Higher? |
| --- | --- | --- | --- | --- |
| GW happening | Closeness | .0007 | .001 | Yes |
| GW human cause | Closeness | .0006 | .001 | Yes |
| GW consensus | Closeness | .001 | .002 | Yes |
| Collective efficacy | Closeness | .001 | .002 | Yes |
| Community risk | Closeness | .0008 | .001 | Yes |
| US risk | Closeness | .0008 | .002 | Yes |
| Risk time | Closeness | .0008 | .001 | Yes |
| General attitude | Closeness | .000009 | .001 | Yes |
| Policy support CO2 | Closeness | .001 | .002 | Yes |
| Policy support fund | Closeness | .001 | .002 | Yes |
| Policy support rebate | Closeness | .002 | .002 | Yes |
| Political behavior | Closeness | .002 | .003 | Yes |
| Consumer behavior reward | Closeness | .002 | .002 | Yes |
| Consumer behavior punish | Closeness | .002 | .003 | Yes |

*Note*. Closeness centrality of worry was compared with that of other variables. Lower and Upper indicate 95% confidence interval of the difference. Significance was tested by comparing the difference score with 0 at the alpha level 0.05.
