# Supplementary material for: Climate change belief systems across political groups in the United States
Source: PLoS One. 2024 Mar 20;19(3):e0300048. doi: 10.1371/journal.pone.0300048 (PMC10954181; doi:10.1371/journal.pone.0300048)
Supplement: S3 Table — (DOCX) [file pone.0300048.s006.docx]

**S3 Table. Strength centrality difference between worry and other elements**

| Variables | Types | Lower | Upper | Significantly Higher? |
| --- | --- | --- | --- | --- |
| GW happening | Strength | .19 | .27 | Yes |
| GW human cause | Strength | .29 | .36 | Yes |
| GW consensus | Strength | .44 | .53 | Yes |
| Collective efficacy | Strength | .67 | .74 | Yes |
| Community risk | Strength | .19 | .27 | Yes |
| US risk | Strength | -.09 | -.005 | No |
| Risk time | Strength | .27 | .35 | Yes |
| General attitude | Strength | .36 | .45 | Yes |
| Policy support CO2 | Strength | .19 | .27 | Yes |
| Policy support fund | Strength | .24 | .32 | Yes |
| Policy support rebate | Strength | .32 | .40 | Yes |
| Political behavior | Strength | .68 | .76 | Yes |
| Consumer behavior reward | Strength | .16 | .24 | Yes |
| Consumer behavior punish | Strength | .09 | .18 | Yes |

*Note*. Strength centrality of worry was compared with that of other variables. Lower and Upper indicate 95% confidence interval of the difference. Significance was tested by comparing the difference score with 0 at the alpha level 0.05.
