# Supplementary material for: Climate change belief systems across political groups in the United States
Source: PLoS One. 2024 Mar 20;19(3):e0300048. doi: 10.1371/journal.pone.0300048 (PMC10954181; doi:10.1371/journal.pone.0300048)
Supplement: S4 Table — (DOCX) [file pone.0300048.s007.docx]

**S4 Table. Difference of GDD scores in intra-group comparison of belief systems**

| Group 1 | Group 2 | Difference Before | Difference After |
| --- | --- | --- | --- |
| Democratic | Republican | -.001 [-.03, .03] | -.001 [-.02, .02] |
| Independent | Republican | .12^***^ [.09, .15] | .12^***^ [.10, .14] |
| No party | Republican | .21^***^ [.18, .24] | .16^***^ [.15, .18] |
| Independent | Democratic | .12^***^ [.09, .15] | .12^***^ [.11, .14] |
| No Party | Democratic | .21^***^ [.19, .24] | .17^***^ [.15, .18] |
| No Party | Independent | .09^***^ [.06, .12] | .04^***^ [.03, .06] |

Note: Tukey method was used for multiple comparisons of GDD means. Difference Before represent difference score estimated before removing outliers and Difference After represent difference score estimated after removing outliers.
