# Supplementary material for: Climate change belief systems across political groups in the United States
Source: PLoS One. 2024 Mar 20;19(3):e0300048. doi: 10.1371/journal.pone.0300048 (PMC10954181; doi:10.1371/journal.pone.0300048)
Supplement: S5 Table — (DOCX) [file pone.0300048.s008.docx]

**S5 Table. Difference of GDD scores in inter-group comparison of belief systems**

| Group 1 | Group 2 | Difference Before | Difference After |
| --- | --- | --- | --- |
| Democratic-No Party | Democratic-Independent | .07^***^ [.04, .09] | .03^***^ [.04, .09] |
| Independent-No party | Democratic-Independent | .13^***^ [.10, .16] | .10^***^ [.08, .11] |
| Republican-Democratic | Democratic-Independent | -.03^**^ [-.06, -.01] | -.03^***^ [-.05, -.02] |
| Republican-Independent | Democratic-Independent | -.03 [-.05, .001] | -.03^***^ [-.04, -.01] |
| Republican-No Party | Democratic-Independent | .10^***^ [.07, .12] | .07^***^ [.05, .08] |
| Independent-No Party | Democratic-No Party | .06^***^ [.04, .09] | .06^***^ [.05, .08] |
| Republican-Democratic | Democratic-No Party | -.10^***^ [-.13, -.07] | -.07^***^ [-.08, -.05] |
| Republican-Independent | Democratic-No Party | -.10^***^ [-.12, -.06] | -.07^***^ [-.07, -.05] |
| Republican-No Party | Democratic-No Party | .03^**^ [.01, .06] | .03^***^ [.02, .05] |
| Republican-Democratic | Independent-No Party | -.16^***^ [-.19, -.14] | -.13^***^ [-.14, -.12] |
| Republican-Independent | Independent-No Party | -.15^***^ [-.18, -.13] | -.12^***^ [-.14, -.11] |
| Republican-No Party | Independent-No Party | -.03^*^ [-.06, -.004] | -.03^***^ [-.05, -.02] |
| Republican-Independent | Republican-Democratic | .01 [-.02, .03] | .01 [-.01, .02] |
| Republican-No Party | Republican-Democratic | .13^***^ [.10, .16] | .10^***^ [.08, .11] |
| Republican-No Party | Republican-Independent | .12^***^ [.10, .15] | .09^***^ [.08, .11] |

Note: Tukey method was used for multiple comparisons of GDD means. Difference Before represent difference score estimated before removing outliers and Difference After represent difference score estimated after removing outliers.
