# Supplementary material for: Climate change belief systems across political groups in the United States
Source: PLoS One. 2024 Mar 20;19(3):e0300048. doi: 10.1371/journal.pone.0300048 (PMC10954181; doi:10.1371/journal.pone.0300048)
Supplement: Attachment — Submitted filename: Response to Reviewer 2.docx [file pone.0300048.s010.docx]

Authors’ Response to Reviewer #2

We appreciate the reviewer's constructive feedback throughout the paper. Please find our itemized responses below. Our responses are italicized.

1. The manuscript lacks technicalities, but the data reflects the Conclusion.

[Authors’ Response] *We appreciate the reviewer’s feedback. To bolster the technical depth of the paper, we have taken into account the suggestions provided in the reviewer’s second comment. Our actions to enhance the manuscript's technicalities are detailed in our response to the second comment provided by the reviewer.*

2. Information on the statistical analysis, including the statistical tool used is sketchy; and needs to be elaborated. For instance, there should be a subheading for Data Analysis. There is need to mention the version of the statistical tool used, including manufacturer, city and country manufactured.

[Authors’ Response] *We appreciate the reviewer’s constructive comments that enhance the quality of the paper. We concur with the reviewer's observation that the original manuscript lacked detail in explaining the analysis and the statistical tool used. As the reviewer suggested, we have added a new section titled “Data Analysis” to provide details about the version of our statistical tool (R 4.3.1) and our data analysis approach (see revised lines 451 to 464 on page 14). Furthermore, we have now included a reference for our statistical tool (R Core Team, 2022), ensuring readers are informed about the manufacturer, city, and country of manufacture.*

*Reference*

*R Core Team. R: A language and environment for statistical computing [Internet]. Vienna, Austria: R Foundation for Statistical Computing; 2022. Available from: https://www.R-project.org/*

3. The study rationale is not properly captured in the 'Introduction'. The identified knowledge gap(s) should be adequately rationalised to maintain a logical flow in the ‘Introduction’.

[Authors’ Response] *We are grateful for the reviewer's feedback, which has helped us to refine the rationale of our study. In response to the suggestion that the identified knowledge gaps require clearer justification, we have increased the detail of our literature review (see revised lines 39 to 42 on page 3). This includes the addition of a study (Verschoor et al., 2020) that applies the belief system framework to the context of climate change, which we have now referenced to bolster the rationale for our research. Moreover, we have added the third paragraph of introduction to better articulate the focus of our study and the significance of our investigation (see revised lines 43 to 66 on page 3 and 4).*

*Reference*

*Verschoor M, Albers C, Poortinga W, Böhm G, Steg L. Exploring relationships between climate change beliefs and energy preferences: A network analysis of the European Social Survey. Journal of Environmental Psychology. 2020 Aug;70:101435.*
